# The Use of Simulation to Improve Family Understanding and Support of Anesthesia Providers

**DOI:** 10.7759/cureus.2262

**Published:** 2018-03-02

**Authors:** Susan M Martinelli, Fei Chen, Gene Hobbs, Brooke A Chidgey, Lacey E Straube, David Zvara, Robert Isaak

**Affiliations:** 1 Department of Anesthesiology, University of North Carolina School of Medicine; 2 Department of Neurosurgery, University of North Carolina School of Medicine

**Keywords:** wellness, simulation, anesthesia, communication, family support

## Abstract

Introduction

Burnout in medical providers is associated with work dissatisfaction, reduction in patient safety, and provider depression. Simulation is a tool effectively used for specific task training but has not been broadly used as a means to combat medical professional stress and enhance wellness. The authors created a medical simulation program targeted at those involved in the social support of medical providers. The hypothesis was that education of non-medical persons involved in social support would translate into an enhanced understanding of the demands among medical providers in anesthesiology. This understanding would thereby open communication pathways within the social support system and contribute to enhanced wellness among providers.

Methods

To assess effectiveness and benefits of the event, survey data were obtained from anesthesia providers and their adult support persons before and after the event. The anesthesia providers were queried on their perception regarding the benefit of the event for their support persons. Support persons were asked questions regarding their understanding of the role of an anesthesia provider.

Results

Sixty-three family members and friends (adult=30, child=33) participated in a two-hour simulation event including activities for participants of all ages. Twenty-nine (96.7%) adult participants (age ≥ 14) completed the support person surveys before and/or after the event. The post-event survey results revealed participants’ satisfaction with the event (n=26, 100%). This simulation event also demonstrated an improved understanding of the demands among anesthesia providers by their support persons (seven items, P values range from less than .0001 to .0313). Most anesthesia providers who attended the event enjoyed it a significant amount (n=19, 82.6%). Most providers whose primary work-related support persons attended the event believed that it would be easier to communicate work-related issues (n=12, 85.7%).

Conclusion

We outline "The Family Anesthesia Experience Day" as a wellness initiative for anesthesia providers. Our study demonstrated improved understanding of support persons’ knowledge about anesthesia providers’ work-related stress via an immersive two-hour simulation-based learning experience. The event was well-received and may be a useful approach to provide support persons with an opportunity to learn about and better support their beloved anesthesia provider.

## Introduction

Strong social support systems, comprised of individuals who understand work-related demands, are an important component of physician well-being [1-2]. Similar to other physicians and healthcare professionals, anesthesiologists’ well-being is intimately connected to the social support received from their family and friends during their professional training and beyond [2-3].

Relatedness is achieved through engaging in meaningful conversation while feeling understood and appreciated by others [1]. However, making time and effort to communicate, while developing and sustaining strong relationships amid demanding careers, can be challenging [2]. When Rappaport et al. surveyed surgery residents’ spouses from 18 different residency programs, 85% of respondents felt their spouse spent more time at work than they had expected prior to the start of residency [3]. Additionally, 69% of respondents expressed resentment towards their spouse over the long hours spent away from home. Almost all (98%) respondents indicated that their spouse’s residency training contributed to at least one negative effect on their marriage. More recently, Law et al. found that residents had difficulty explaining their job to those outside of medicine and felt that their family and friends needed to change their expectations of them as work-related obligations were frequently their first priority [4]. Non-anesthesia providers' understanding of the role of an anesthesia provider and the accompanied work-related demands may be even less than that of other medical subspecialties, as research showed that only 40% to 88% of patients knew that an anesthesiologist was a physician [5]. Most patients don’t understand the role of an anesthesiologist after the patient has been induced, or that anesthesiologists serve in roles outside of the operating room [5].

This article outlines "The Family Anesthesia Experience Day", a wellness initiative in an anesthesiology department that employs the novel use of simulation. The purpose of this program is to bridge the communication divide between what the provider experiences at work and what is understood by those who form the support system for the provider outside of work. Through this simulation-based event, we provided an experiential learning opportunity for spouses, partners, parents, and children to see what their loved one experiences in the workplace with the hope that the insight gained will open a new channel of communication, allowing for better understanding and greater support between the anesthesia provider and his/her support persons.

## Materials and methods

The project presented was reviewed by the Office of Human Research Ethics of University of North Carolina at Chapel Hill, which has determined that the activity does not constitute human subjects research as defined under federal regulations [45 CFR 46.102 (d or f) and 21 CFR 56.102(c)(e)(l)] and does not require IRB approval.

We developed an immersive, in-person, two-hour event held in our simulation center focused on the details surrounding the patient-care activities of anesthesiology. All anesthesia providers [residents, attendings, and certified registered nurse anesthetists (CRNAs)] were encouraged to invite their support persons to participate in the event. As we had limited resources and space, we had to limit the number of participants. The attendance was on a first come, first serve basis through electronic sign-up.

Initially, all of the participants gathered in a briefing room for a welcome and introduction to the goals of the event. The primary purpose of the event was to provide the participants with an understanding of the typical demands on an anesthesia provider while taking into account the stress and unpredictability of daily events.

**Figure 1:**
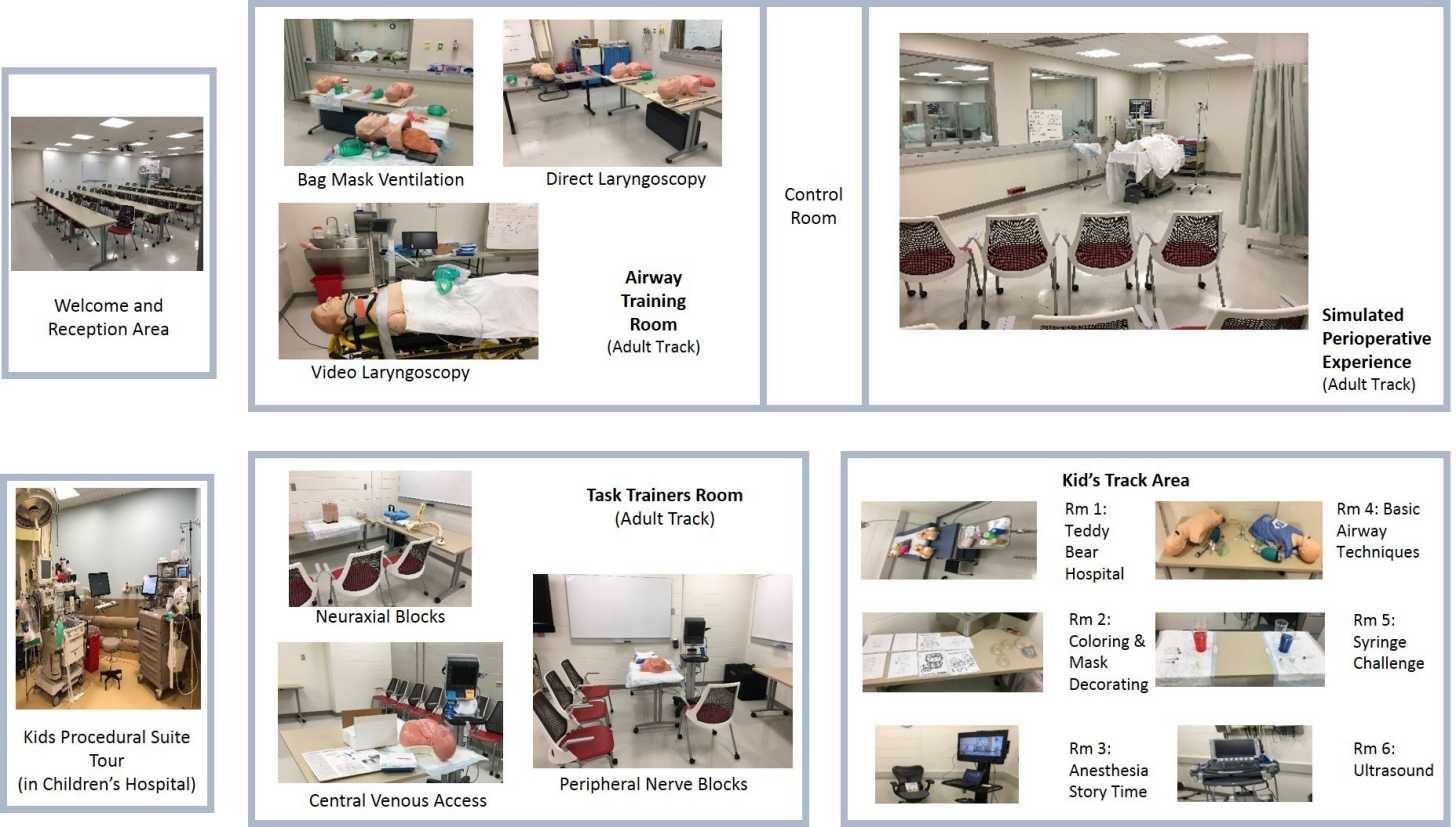
Simulation space layout The diagram depicts the layout of the simulation center for the adults' and kids' tracks. The photographs provide the equipment and orientation of the stations. Participants were evenly divided amongst the stations to start and then were directed to rotate to each station within their track at designated times provided by the program moderator.

Two parallel tracks were developed and ran simultaneously. (See Figure 1 for details of the layout.) One of the tracks was a “kid’s track” geared toward three- to ten-year-olds. The other was an “adult track” intended for middle school-aged participants and up. Those participating in the “kid’s track” were split into small groups and partook in multiple stations to introduce children to the medical field, specifically anesthesiology. These stations included basic airway management (e.g. placement of a facemask on a mannequin), utilization of an ultrasound to see blood vessels and nerves on themselves, a teddy bear hospital to repair “injured” stuffed animals, arts and crafts (e.g. medical themed coloring pages and face mask decorating), and a tour of a real procedural suite in the children’s hospital. As the kids ranged from 3-11 years old, they were not surveyed due to the wide range inability to comprehend survey questions that would have been comprehensive enough to provide meaningful results.

For the adult track (ages 14 and up), participants split into three groups and rotated through three core stations. The first core station consisted of a simulated patient experience utilizing standardized patient actors, real anesthesia clinicians (attending anesthesiologist and CRNA) and a high-fidelity patient simulator (Laerdal SimMan3G, Wappinger Falls, NY). The participants observed a live preoperative patient assessment, a TeamSTEPPS (Team Strategies and Tools to Enhance Performance and Patient Safety) preoperative briefing with a simulated surgeon and operating room (OR) nurse, induction of anesthesia, an intraoperative code, and a verbal patient handoff [e.g. situation, background, assessment, recommendation (SBAR)] to an intensive care unit physician over the telephone. The participants then took part in a debriefing discussion of the scenario that was facilitated by an attending anesthesiologist. The debriefings served to engage the participants in a discussion regarding the various responsibilities of an anesthesia provider and the resultant stressors that ensue for this career. As is fairly common in simulation debriefings, there were no set discussion objectives. Rather, the debriefings were facilitated discussions based on what impacted the participants the most when they observed the scenario. The debriefings included both support persons and anesthesia providers, which provided for an engaging conversation and opportunities for clarification regarding the anesthesia-related tasks that were observed in the simulation. The simulation debriefings were not intended to provide specific training on how support persons could provide emotional support, but instead an opportunity to observe and discuss an intense anesthetic-related crisis. In the other two core stations, the groups of participants divided into smaller groups for a more hands-on experience. Each of these core stations had three substations focused on various anesthesia skills that gave participants an opportunity to perform procedures on simulated task trainers. One core station consisted of partial task trainers for the placement of epidurals, central lines, and peripheral nerve blocks. The other core station exposed the participants to airway management, specifically bag-mask ventilation, direct laryngoscopy, and video laryngoscopy.

In order to assess the effectiveness and benefits of the event, survey data were obtained from anesthesia providers and their adult support persons (ages 14 and older) both before and after the event. Specifically, the providers and participants were asked attitudinal questions pertaining to the event. In addition to gathering demographic information, participants were asked questions regarding their understanding of the role of an anesthesia provider. The anesthesia providers were surveyed on their perception regarding the benefit of the event for family and friends.

## Results

We obtained responses from 29 of the 30 (96.7%) adult support persons. Twenty-five (86.2%) of the 29 support persons responded to the pre-survey, among which 21 (72.4%) were complete. Twenty-six support persons completed the post-survey. We matched a total of 22 (75.9%) paired pre- and post-responses, among which 21 (72.4%) were complete. See Table 1 for demographics of the support persons.

**Table 1:** Family member participant demographics (n=29) Note: NA=Not available due to missing value, CRNA=Certificated Registered Nurse Anesthetist *For those who answered yes, we asked for more information on their occupation: 1 physical therapist, 1 EMT/ED technician, 1 nursing student, 1 nurse, 1 licensed athletic trainer, and 1 did not provide an answer.

Demographics	Level	No. (%)
Gender	Female	15 (51.7)
	Male	11 (37.9)
	NA	3 (10.3)
Invited by	Resident	10 (34.6)
	Attending	16 (55.2)
	CRNA	3 (10.3)
Relationship	Spouse/Significant other	14 (48.3)
	Anesthesia provider is my parent	2 (6.9)
	Anesthesia provider is my child	8 (27.6)
	Other (Sibling, friend, etc.)	5 (17.2)
Personal experience providing health care	Yes*	6 (20.7)
	No	16 (55.2)
	NA	7 (24.1)
Distance traveled to attend the event	Live together	16 (55.2)
	Within 30min	2 (6.9)
	Within 2 h	6 (20.7)
	Within 8h	1 (3.4)
	More than 8h	1 (3.4)
	NA	3 (10.3)

Participant appraisal of the event

All responding support persons felt they learned a moderate to significant amount about the daily experiences of an anesthesia provider from the event. All responders stated that they enjoyed participating in the event and would recommend this event to others. Most (n=19, 82.6%) anesthesia providers who attended the event enjoyed it a significant amount. Most providers whose primary work-related social support person attended the event believed that their well-being would be improved by a moderate (n=11, 44.0%) or significant amount (n=8, 32.0%), and agreed that it would improve work-related communication with their social support person (n=12, 85.7%). (See Table 2 for the anesthesia provider’s full appraisal of the event.)

**Table 2:** Anesthesia providers’ appraisal of the event

Question	Responder	Answer	No. (%)
How much did you enjoy participating in this event?	Only providers who attended the event as volunteers (n=23)	A significant amount	19 (82.6)
		A moderate amount	3 (13.0)
		A minimal amount	1 (4.3)
		Not at all	0 (.0)
How much, if at all, do you think your family member or friend's participation in this event will help to improve your overall well-being?	Only providers who have at least one family member attended the event (n=25)	A significant amount	8 (32.0)
		A moderate amount	11 (44.0)
		A minimal amount	5 (20.0)
		Not at all	0 (.0)
		I don't know	1 (4.0)
After this event, how much, if at all, do you think this person understands the stresses involved in practicing anesthesia?	Only providers whose primarily work-related emotional support attended the event (n=14)	Completely	2 (14.3)
		Mostly	9 (64.3)
		A little	3 (21.4)
		Not at all	0 (.0)
After this event, how much, if at all, do you think your well-being will be improved as the result of this person attending the event?	Only providers whose primarily work-related emotional support attended the event (n=14)	Completely	1 (7.1)
		Mostly	6 (42.9)
		A little	7 (50.0)
		Not at all	0 (.0)
After this event, it will be easier for me to communicate my work-related issues with this person.	Only providers whose primarily work-related emotional support attended the event (n=14)	Strongly agree	4 (28.6)
		Somewhat agree	8 (57.1)
		Neither agree nor disagree	2 (14.3)
		Somewhat disagree	0 (.0)
		Strongly disagree	0 (.0)

Family members’ perception of knowledge improvement

**Figure 2:**
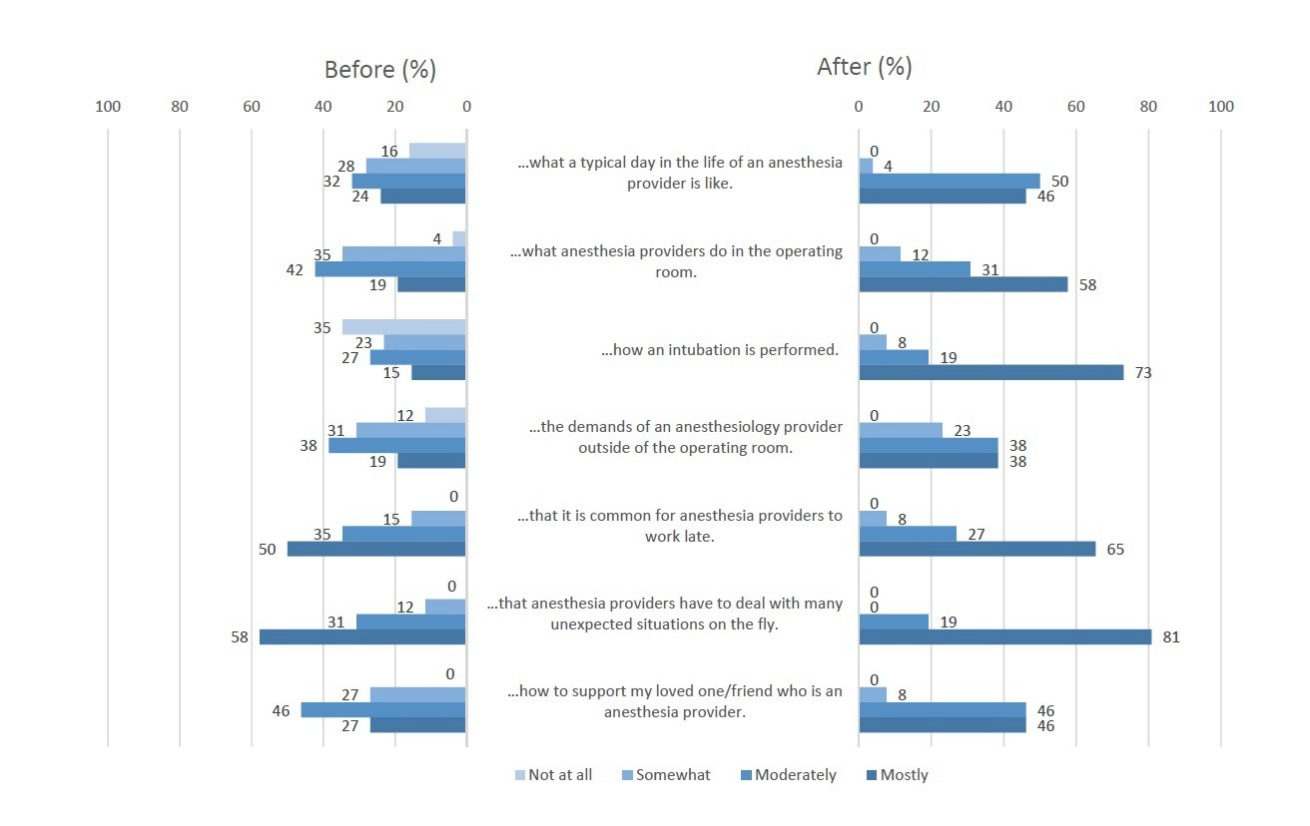
Family member and friend participants’ perceived understanding of anesthesia providers’ work before and after the event (N=26) *Note:* One missing answer to the pre-event survey question on “I understand what a typical day in the life of an anesthesia provider is like”.

In both the pre- and post-surveys, we included seven statements pertaining to anesthesia providers’ work-related demands for their support persons to rate regarding their perceived knowledge. Additionally, in the post-survey, we asked the support persons to reflect on the anesthesia knowledge they had prior to the event. Wilcoxon signed-rank tests examined the difference between the self-reported perception of knowledge before attending the event, collected in the pre-event survey, and the reflection of knowledge prior to the event, collected in the post-event survey. No statistically significant difference was found between support persons’ initial assessment of their prior knowledge and the re-assessment of their prior knowledge of any knowledge item. Thus, given that more responses from the post-survey were complete, we compared the difference between the self-reported perception of the knowledge after attending the event and the re-assessment of prior knowledge to examine the change in support persons’ perceived understanding of what anesthesia providers do at work. The results of Wilcoxon signed-rank test found a statistically significant increase in perceived knowledge on all seven items surveyed (Table 3). As depicted in Figure 2, before the event, all support persons understood it is common for anesthesia providers to work late. However, only 58% of the support persons “mostly” understood that anesthesia providers have to deal with many unexpected situations on the fly and 19% “mostly” understood what anesthesia providers do in the operating room and the demands of an anesthesia provider outside of the operating room. After the event, 81% of the support persons “mostly” understood the demands of dealing with unexpectedness involved in anesthesia providers’ work; 58% “mostly” understood the demands in the operating room; 38% “mostly” understood the demands outside of the operating room. In addition, all support persons indicated that they had at least some understanding of a typical day in the life of an anesthesia provider after the event.

**Table 3:** Change in family members' and friend participants’ perceived understanding of anesthesia providers’ work ^a ^The answers were given on an ordinal rating scale (0 = not at all, 1=Somewhat, 2=Moderately, and 3=Mostly). ^b ^S=Signed Rank: Difference=post assessment – reflection of prior knowledge. Post assessment stands for the self-reported perception of the knowledge after attending the event. Reflection of prior knowledge stands for the re-assessment of knowledge prior to the event informed with the knowledge acquired in the event. ^c^P value based on Wilcoxon signed-rank test of median difference equal to zero.

Item^a^	N	S^b^	P value^c^
I understand…			
…what a typical day in the life of an anesthesia provider is like.	25	68	<.0001
…what anesthesia providers do in the operating room.	26	45.5	.0002
…how an intubation is performed.	26	105	<.0001
…the demands of an anesthesiology provider outside of the operating room.	26	58.5	.0023
…that it is common for anesthesia providers to work late.	26	10.5	.0313
…that anesthesia providers have to deal with many unexpected situations on the fly.	26	22.5	.0039
…how to support my loved one/friend who is an anesthesia provider.	26	22.5	.0039

## Discussion

Our study demonstrated improved understanding of support persons’ knowledge about anesthesia providers’ work-related demands via an immersive two-hour simulation-based learning experience. This simulation-based family educational event was well-received by our department. The anesthesia providers’ support persons who attended enjoyed the event, reported increased knowledge of the role of an anesthesia provider, and would recommend the event to others. The anesthesia providers felt that they would be better able to communicate with their support persons who attended the event and felt that the event would increase their personal well-being.

The emphasis on well-being for healthcare providers is gaining a significant amount of importance in the medical arena. In July 2017, the Accreditation Council for Graduate Medical Education (ACGME) adapted the Core Program Requirements for residency programs to include an obligation to implement policies that increase the well-being of both residents and their faculty. Additionally, resident well-being is emphasized in the Anesthesiology Milestone Project with an entire Professionalism sub-competency milestone (Professionalism 5: Responsibility to maintain personal emotional, physical, and mental health) dedicated to this topic [6]. This event is a targeted opportunity to increase wellness for our providers. Increasing wellness is one strategy to combat burnout amongst anesthesiology providers. The prevalence of burnout is increasing for physicians (45.5% in 2011 to 54.4% in 2014), while satisfaction with work-life balance is decreasing (48.5% in 2011 to 40.9% in 2014) [7]. Anesthesiologists, in particular, have a higher rate of burnout and lower satisfaction with work-life balance than average physicians [7-8]. Our findings suggest that support persons likely don’t have a good understanding of what happens in the perioperative environment, including the emotional stresses and unpredictability of anesthesia-related work. We believe that this lack of understanding may lead to a decreased ability to support an anesthesia provider after a stressful event occurs in the clinical environment. In addition, it could be challenging to discuss a success in the operating room with someone who does not have the baseline knowledge of the role of an anesthesia provider. Thus, investigations are warranted into whether an improved understanding of anesthesia providers’ work and work-related demands by social support persons serves as a buffer for burnout and improves psychological well-being.

This initial study demonstrated that this event could be successfully carried out from a logistics standpoint. We were able to gather baseline data that demonstrated an increase in support persons’ knowledge of what an anesthesia provider does. However, our current data is limited to the immediate post-event responses of the participants without investigating any long-term effects of the program or effects on clinician well-being. Future studies should consider collecting data at multiple time points following the event. It may also be prudent to directly examine the effects the event has on additional areas of our clinicians’ well-being, such as stress, burnout, and resilience, as well as the anesthesia providers’ perception of personal well-being and improved support person understanding of their work. Additionally, the current event solely provided a simulation experience without any background information on specific challenges that our clinicians may face. Future events may incorporate didactic components on well-being, burnout, substance abuse, and financial well-being. Lastly, this study was conducted within a single department at a single institution. Greater generalizability may come from work done at multiple institutions. Furthermore, it may also be warranted to test the utility of such a wellness event in a variety of anesthesiology departments (both academic and private) as well as a number of other procedurally and high-stakes based specialties (e.g. surgery or emergency medicine) to aid in promoting wellness. This program may also be expanded to medical students and a variety of other healthcare professionals. This wellness initiative was relatively inexpensive to conduct, was embraced with enthusiasm, and enhanced the understanding of the work environment in those who provide social support for our medical caregivers.

## Conclusions

Improved understanding and relatedness between anesthesia providers and their support persons is a key factor in promoting their wellness. We designed and implemented an innovative simulation-based educational program to help support persons understand and support anesthesia providers’ work-life (including stressors, tasks, and responsibilities) via an experiential learning approach. Our survey data showed that the event was well-received and may be a useful approach to provide support persons with an opportunity to learn, better understand, and support their beloved anesthesia providers’ navigation in the challenging demands of balancing a career and personal life.
